# Mental Health in the Consorci Sanitari del Maresme workers during the COVID-19 pandemic: A descriptive cross sectional study

**DOI:** 10.1192/j.eurpsy.2022.1362

**Published:** 2022-09-01

**Authors:** M. González-Campos, O. Brugué González

**Affiliations:** Hospital de Mataró, Pshychiatry Department, Barcelona, Spain

**Keywords:** mental health, Covid-19 pandemic, Mental Disorders, healthcare workers

## Abstract

**Introduction:**

The global health emergency caused by the COVID-19 pandemic has put healthcare professionals in an unprecedented challenge, considering them extremely at risk population.

**Objectives:**

To estimate the prevalence of clinically significant mental disorders and to assess associated factors among Consorci Sanitari del Maresme workers during the COVID-19 pandemic.

**Methods:**

We made a descriptive cross-sectional study. All workers were invited to participate in an online survey during May 2021. Individual characteristics and frequency of direct exposure to COVID during professional activity were assessed. We used three Spanish versions of psychometric scales: the Patient Health Questionnaire to evaluate depressive symptomatology, the Generalized Anxiety Disorder scale, which detects anxiety, and the 4-item version of the PTSD checklist for DSM-5 for PTSD screening purposes. Chi-square tests and logistic regression were used to analyze the data.

**Results:**

A total of 355 workers participated. Overall, 31% met the criteria for Major Depressive Disorder (PHQ-9> 9), 36% for Generalized Anxiety Disorder (GAD-7> 9) and 22% for Post-traumatic Stress Disorder (PCL-5> 7). It has been found that young adult, women, those with prior mental disorders or those with greater exposure to COVID-19 are risk factors for any current mental disorder.

**Conclusions:**

There are large mental healthcare needs among healthcare professionals. There is a clear need to closely monitor the extent to which these needs are adequately met. In the design of measures and interventions to reduce this impact, an individualized approach should be considered while taking into account sociodemographic variables, psychiatric history and the frequency of direct exposure to COVID-19.

**Disclosure:**

No significant relationships.

